# SADLN: Self-attention based deep learning network of integrating multi-omics data for cancer subtype recognition

**DOI:** 10.3389/fgene.2022.1032768

**Published:** 2023-01-04

**Authors:** Qiuwen Sun, Lei Cheng, Ao Meng, Shuguang Ge, Jie Chen, Longzhen Zhang, Ping Gong

**Affiliations:** ^1^ School of Medical Imaging, Xuzhou Medical University, Xuzhou, China; ^2^ School of Information and Control Engineering, University of Mining and Technology, Xuzhou, China; ^3^ Department of Radiation Oncology, Affiliated Hospital of Xuzhou Medical University, Xuzhou, China

**Keywords:** self-attention, deep learning, multi-omics data, Gaussian mixture model, cancer subtype recognition

## Abstract

Integrating multi-omics data for cancer subtype recognition is an important task in bioinformatics. Recently, deep learning has been applied to recognize the subtype of cancers. However, existing studies almost integrate the multi-omics data simply by concatenation as the single data and then learn a latent low-dimensional representation through a deep learning model, which did not consider the distribution differently of omics data. Moreover, these methods ignore the relationship of samples. To tackle these problems, we proposed SADLN: A self-attention based deep learning network of integrating multi-omics data for cancer subtype recognition. SADLN combined encoder, self-attention, decoder, and discriminator into a unified framework, which can not only integrate multi-omics data but also adaptively model the sample’s relationship for learning an accurately latent low-dimensional representation. With the integrated representation learned from the network, SADLN used Gaussian Mixture Model to identify cancer subtypes. Experiments on ten cancer datasets of TCGA demonstrated the advantages of SADLN compared to ten methods. The Self-Attention Based Deep Learning Network (SADLN) is an effective method of integrating multi-omics data for cancer subtype recognition.

## 1 Introduction

Cancer is one of the most common and fatal diseases with high heterogeneity, that is same cancer will produce subtypes with different phenotypes, which will affect the clinical treatment and prognosis (Bray et al., 2018; Siegel et al., 2020). Therefore, the recognition of the cancer subtype is of great significance for the choice of treatment and prognosis of cancer patients (Hong Zhao et al., 2014). With the developments of high-throughput sequencing technology, there yield large amounts of multi-omics data, such as miRNA expression data, mRNA expression data, DNA methylation data, and copy number variation etc. (Song et al., 2020). These multi-omics data can be obtained by some publicly available projects. For example, The Cancer Genome Atlas (TCGA) (Sayáns et al., 2019) stories more than 30 cancers over 11,000 patients’ data and provides valuable opportunities for cancer subtype recognition. Existing studies have demonstrated that incorporating multi-omics data can obtain better performances and improve the understanding of cancer progression compared to using single-omic data (Hawkins et al., 2010; Kristensen et al., 2014; Hasin et al., 2017). Therefore, there is a strong need for integrated analysis of multi-omics data in cancer subtype recognition (Simidjievski et al., 2019; Xu et al., 2019; Picard et al., 2021).

The clustering algorithm is often used to recognize cancer subtypes. Researchers have proposed many clustering methods for multi-omics data integration. These methods can be divided into three categories: early integration, late integration, and intermediate integration (Rappoport and Shamir, 2018).

Early integration methods simply concatenate different omics’ feature matrices to a single matrix and use the single omics clustering algorithm to subtype the matrix (Rappoport and Shamir, 2018). For example, K-means, LRAcluster, and Spectral clustering all belong to this category. Early integration methods do not consider the differences in the distribution and information contribution of each omics data, they increase the dimension of input data and exacerbate the dimension problem. In late integration, each omic data is clustered separately and the clustering solutions are integrated to obtain a single clustering solution. For example, COCA (Le et al., 2016) and PINS (Nguyen et al., 2017) belong to this category. Late integration methods ensure robustness against noise and bias, but the performance may be greatly affected when each omics data have different degrees of information contribution.

On the other hand, intermediate integration attempts to build a model that integrates all omics, including the method of integrating sample similarity, the method of using joint size reduction, and the method of using data statistical modeling. Similarity-based ensemble methods construct and fuse the sample similarity at each omics level to obtain consistent sample-sample relationships, and then perform cluster analysis. Typical methods include SNF (Wang et al., 2014) and NEMO (Rappoport and Shamir, 2019). These methods are very sensitive to data noise or network parameters due to the instability of the kernel distance function. An ensemble method based on dimensionality reduction is used to project each omics data into a common low-dimensional space, typical methods are CCA and MCCA (Witten and Tibshirani, 2009). However, these methods are susceptible to data noise and feature heterogeneity. Statistics-based ensemble methods build a statistical model to tackle ensemble challenges, including cluster (Shen et al., 2009), iClusterPlus and iClusterBayes.

As machine learning development, deep learning has been widely used in healthcare, such as imaging-based computer-aided diagnosis (Yu et al., 2021), digital pathology (Parodi et al., 2015), drug design (Peng et al., 2020), prediction of hospital admission (Zhang et al., 2022), classification of cancer (Zeng et al., 2021), and so on. With the advancement of the high learning capability and flexibility of deep neural networks, more and more deep learning based multi-omics integration methods have been proposed for cancer subtype recognition (Poirion et al., 2018; Guo et al., 2019). Most of them adopted autoencoder (AE) architecture, such as multi-omics autoencoder integration (MAUI) (Song et al., 2021), stacked sparse autoencoder (SSAE) (Xu et al., 2016), denoising autoencoder for accurate cancer prognosis prediction (DCAP) (Chai et al., 2021), which can efficiently leverage multi-omics datasets to learn latent factors of observed data in lower dimensions. However, these methods are almost based on early integration and ignore the distributions of different omics which would underestimate heterogeneous omics data (Wang et al., 2020). To solve these problems, some researchers have proposed deep learning based middle integration methods (Sharifi-Noghabi et al., 2019; Adossa et al., 2021; Picard et al., 2021). These methods separately learned each omics data through some subnetwork, and then integrated the output of every sub-network into a unified representation. For example, Tong et al. (2020) proposed ConcatAE, a method of concatenating features learned from each omics using an autoencoder. Yang et al. (2021a) proposed Subtype-GAN, an approach that used multi-input multi-output neural networks separately to model multi-omics data. Although these methods have demonstrated good performance in cancer subtype recognition, they ignore the relationship between samples when learning valuable feature representation. Different omics data types could provide unique characteristics to the patients’ space. Therefore, it is crucial to utilize the relationship of patients to further boost learning performance.

More recently, attention mechanism has become a new technology in the field of deep learning. The dominant thought is to measure the similarity between the Key and the Query (Mercer and Neufeld, 2021). Attention mechanism has been applied in speech NLP, image and other fields (Luo et al., 2018; Yuan et al., 2018; Li et al., 2020a; Liu et al., 2020), since it can select the most informative features of an input, adaptively consider the importance of a single feature and allow the model to make a more accurate judgment. As a special, self-attention (Shaw et al., 2018; Hou et al., 2019), which calculates the response at a position in the sequence by attending to all positions within the same sequence has achieved notable success in modeling complicated relations (Gao et al., 2019). For instance, it displays the superiority in machine translation (Zhang et al., 2020), sentence embedding (Li et al., 2020b) of modeling arbitrary word dependency and has been successfully applied to capture node similarities in graph embedding (Mustafa Abualsaud, 2019). Research shows that the attention-based encoder is more fit for learning high-level features (Chen et al., 2021).

To this end, we proposed SADLN: a self-attention based deep learning network integrating multi-omics data for cancer subtype recognition. SADLN is a middle integration method by consolidates the adversarial generation network and the self-attention mechanism to describe the different distributions of multi-omics data and fusion samples’ relationship. It used an independent sub network to learn omics-specific features and concatenated omics-specific features to an integration representation. Then used a self-attention to learn the relationship of samples on the integration representation and obtained a feature representation that fused the sample relationship. Finally, it used the Gaussian Mixture Model (GMM) to obtain the subtyping label of each sample.

The main contribution is summarized as follows:

1) We proposed a novel deep learning method, SADLN, which combines encoder, self-attention, decoder, and discriminator into a unified framework. It can simultaneously integrate multi-omics representation and sample relations.
2) We firstly introduced the self-attention into the deep learning based method for the cancer subtyping recognition task which allows the model to automatically learn the similarity of samples for better representation.
3) We conducted experiments on ten cancer datasets of TCGA, and SADLN achieved outstanding performance compared with ten integration methods. It provided the theoretical basis and a new method for clinical diagnosis and precise treatment of cancer, which has great theoretical significance and clinical application value.

## 2 Methodology

**FIGURE 1:**
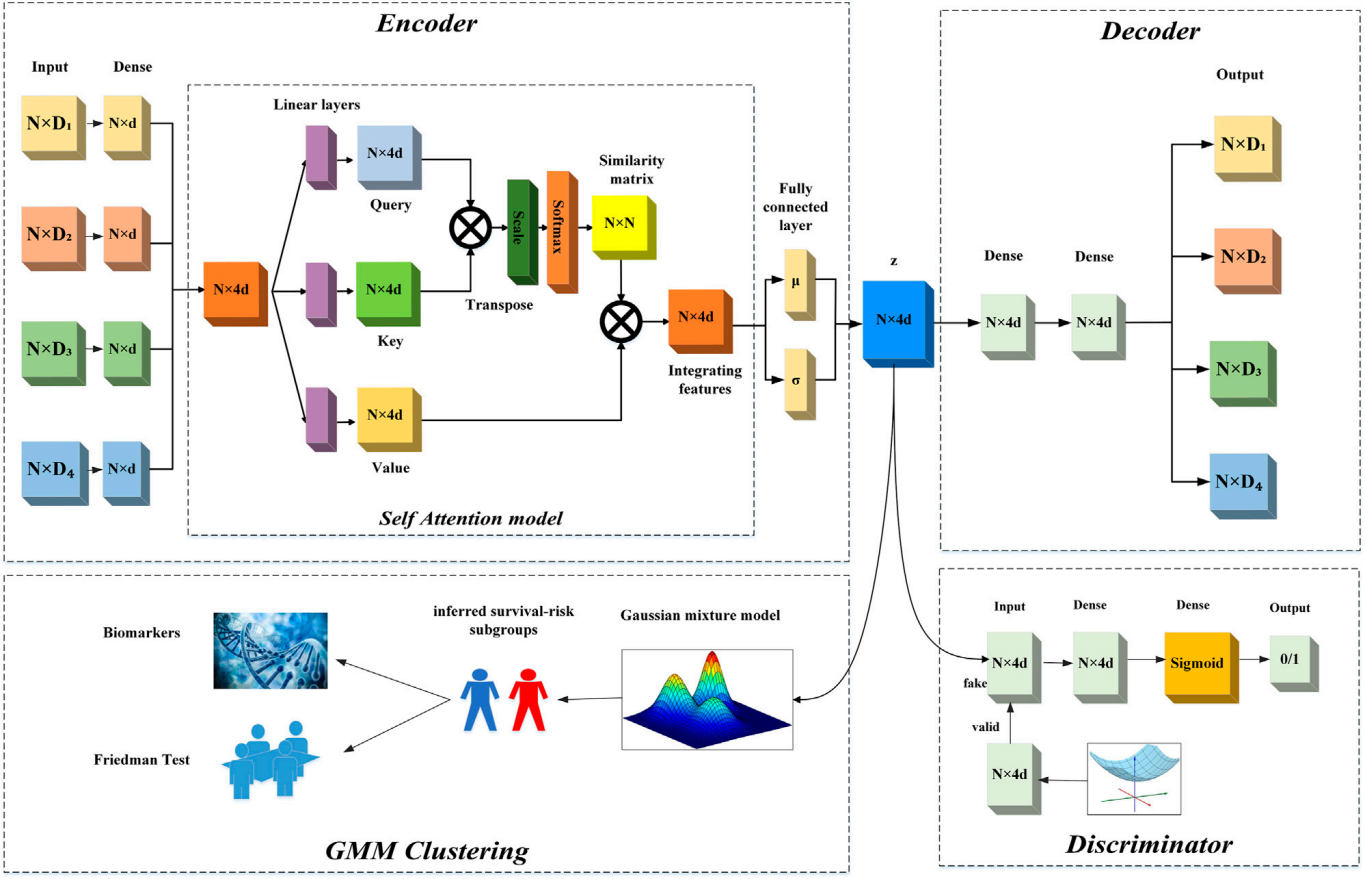
The overview architecture of SADLN.

Our proposed method consists of two steps. Firstly, we used the SADLN model to learn an integrated feature representation from multi-omics data. Secondly, with the learned feature representation, we used the GMM to identify sample’s subtypes. In the SADLN model, the input is the sample’s multi-omics data and the output is the sample’s integrated low-dimensional feature representation. The model consists of three main blocks: self-attention based encoder, decoder and discriminator. Figure 1 gives the overview architecture of our proposed method. In the following, we describe each block in more detail.

### 2.1 Self-attention based encoder

To be able to generate higher quality data distribution, we design a self-attention based encoder in our SADLN model as shown in Figure 1. The self attention based encoder transforms the multi-omics data into a low-dimensional latent space representation *z* with distribution **N**(*μ*, *σ*) using multiple independent network layers, a fully connected layer and the self attention layer. We used four sub-independent dense network to extract features from each original omics data. For each sub-independent layer, let $x^{m}=\left\{x_{1}^{m},\ldots ,x_{N}^{m}\right\}\in R^{N\times D}$ denotes the input of the network for the *m*-th omics data, $y^{m}=\left\{y_{1}^{m},\ldots ,y_{N}^{m}\right\}\in R^{N\times d}$ denotes the output of the *m*-th omics through the sub-independent layer, where *N* is the number of data samples, *D* and *d* are the feature dimension of the input data and the output data respectively. **y** ^*m*^ can be express as:

$$y^{m}=w_{m}x^{m}+b^{m}$$ (1)

where **w**_*m*_ is the weight matrix, **b**_*m*_ is the bias.

To fusion features from different omics data, we concatenate four features matrices into a feature representation matrix. The integrating feature matrix **Y** can be expressed as:

$$Y=Concat\left(y^{1},\ldots ,y^{4}\right)$$ (2)

For example, if the outputs of the sub-networks is a *N* × *d* feature matrix, after concatenation, the output will be one *N* × 4*d* feature representation matrix. To prevent the model overfitting, we appended batch normalization layers and used the Gaussian Error Linear Unit (GELU) function as the non-linear activation function. That is:

$$Y^{\prime }=GELU\left(Y\right)$$ (3)

Although the concatenation operation can integrate multi-omics data, the relationship between samples is not considered. In this study, we introduced self-attention mechanism to construct the relationship between samples. Self-attention is typically used to model the relationship of words in a sentence, we treat each sample’s features vector as a word and learn the samples’ weight matrix through the sample’s feature vectors.

Let *d*_*k*_ = 4*d*, $K=\left[k_{1},k_{2},\ldots ,k_{N}\right]\in R^{N\times d_{k}}$ is a set of keys, $Q=\left[q_{1},q_{2},\ldots ,q_{N}\right]\in R^{N\times d_{k}}$ is a set of queries, $V=\left[v_{1},v_{2},\ldots ,v_{N}\right]\in R^{N\times d_{k}}$ is a set of values, **K** = **Q** = **V** = **Y**′, **K** = **Y**′**W** ^*K*^, **Q** = **Y**′**W** ^*Q*^, **V** = **Y**′**W** ^*V*^. **W** ^*K*^, **W** ^*Q*^, **W** ^*V*^ are the parameters of linear projection layers. $Z=\left\{z_{1},z_{2},\ldots ,z_{N}\right\}\in R^{N\times d_{k}}$ denotes the finally integrating representation, the *j*th feature vector *z*_*j*_ is computed as the following steps (Yang et al., 2021b). Firstly, we use the dot-product between *q*_*i*_ and *k*_*j*_ to compute the similarity of the sample *i* and *j*. To ensure the result does not get excessively large, we scale it by $\sqrt{d_{k}}$ . That is:

$$r_{i,j}=\frac{q_{i}\times k_{j}^{T}}{\sqrt{d_{k}}}$$ (4)

Secondly, softmax function was used to obtain the similarity weight. That is:

$$\omega_{i}=softmax\left\{\frac{q_{i}\times k_{1}^{T}}{\sqrt{d_{k}}},\frac{q_{i}\times k_{2}^{T}}{\sqrt{d_{k}}},\ldots ,\frac{q_{i}\times k_{N}^{T}}{\sqrt{d_{k}}}\right\}$$ (5)

Thirdly, the integrated feature vector *z*_*i*_ of sample *i* can be obtained by a weighted sum of the values. That is:

$$z_{i}=Attention\left(q_{i},K,V\right)=\overset{N}{\underset{j=1}{\sum }}\omega_{i}v_{j}$$ (6)

Finally, the integrated feature representation can be express as:

$$Z=Attention\left(Q,K,V\right)=\left[z_{1},z_{2},\ldots z_{N}\right]\in R^{N\times d_{k}}$$ (7)

To keep the data distribution unchanged, we added batch normalization layers after the self-attention model.

Suppose **Z** obeys Gaussian distribution **Z** ∼ *N*(*μ*, *σ* ^2^), where *μ* is the mean and *σ* ^2^ is the variance. In this paper, we obtained *μ* and *σ* ^2^ through two fully-connected layers.

### 2.2 Decoder

Decoder, in our SADLN model attempts to reconstruct the original multi-omics data from the integrating representation **Z**. As shown in the upper right halves of Figure 1, it contains fully connected layers and an output layer. Let $X_{I}=\left\{x_{I}^{1},x_{I}^{2},x_{I}^{3},x_{I}^{4}\right\}$ denotes the input of encoder, $X_{O}=\left\{x_{O}^{1},x_{O}^{2},x_{O}^{3},x_{O}^{4}\right\}$ denotes the output of decoder. To minimize the error between the input **X**_*I*_ and the output **X**_*O*_ (Badrinarayanan et al., 2017), the square Euclidean distance was applied to calculate the loss *L*_*Decoder*_, it can be expressed as:

$$L_{\mathrm{Decoder}}=\|X_{I}-X_{O}{\|}_{2}^{2}=\frac{1}{4}\overset{4}{\underset{k=1}{\sum }}\|x_{I}^{k}-x_{O}^{k}{\|}_{2}^{2}$$ (8)

### 2.3 Discriminator

To force the distribution of the integrated feature representation matches the prior Gaussian distribution, we added a discriminator D to the model, which is a part of the GAN network. A typical GAN network is composed of a generator G and a discriminator D. In this work, we regard the self-attention base encoder part as the generator G, the input of the discriminator D is the output of the encoder part, and the randomly sampled data with Gaussian distribution. Let *G*(*z*) denote the function of the generator, and *P*(*z*) denote the prior Gaussian distribution. The discriminator D is used to distinguish the samples from *P*(*z*) or the *G*(*z*) (Yang et al., 2021a). Through adversarial learning, *G*(*z*) is as close to *P*(*z*) as possible.

The objective function optimization of discriminator D adopts the method of maximization and minimization. It can be expressed as:

$$\underset{G}{\min}\underset{D}{\max}E_{z^{\prime }∼P\left(z\right)}\left[logD\left(z^{\prime }\right)\right]+E_{z∼G\left(z\right)}\left[log\left(1-D\left(z\right)\right)\right]$$ (9)

where *E* represents the expected value of the distribution function. We use the binary_crossentropy function to train the discriminator learning process. The loss of the discriminator is:

$$L_{\mathrm{Discr}}=-E_{z^{\prime }∼P\left(z\right)}\left[logD\left(z^{\prime }\right)\right]-E_{z∼G\left(z\right)}\left[log\left(1-D\left(z\right)\right)\right]-E_{z∼G\left(z\right)}\left[log\left(D\left(z\right)\right)\right]$$ (10)

Our model parameters of the whole network are jointly trained by minimizing the following total loss:

$$L=\lambda_{1}L_{\mathrm{Decoder}}+\lambda_{2}L_{\mathrm{Discr}}$$ (11)

where *L*_*Decoder*_ and *L*_*Discr*_ are defined in Eq.8 and Eq. 11, respectively. *λ*_1_ and *λ*_2_ ∈ [0, 1] are trade-off parameters.

### 2.4 The GMM clustering of SADLN

For the generated feature representation $Z={\left\{z_{n}\right\}}_{n=1}^{N}$ , we use GMM to identify sample’s subtypes. GMM is a probabilistic clustering method, which also belongs to the generative model. It assumes that all the data points are generated from a mixture of a finite number of Gaussian distributions (Gu et al., 2020). GMM model has excellent clustering performance. In this paper, we use GMM as the clustering module. Let *K* denotes the number of clusters, *π* = (*π*_1_, *π*_2_, …, *π*_*k*_) represent the weight of each cluster, *μ* = (*μ*_1_, *μ*_2_, …, *μ*_*k*_) is the mean vector, *∑* = (*∑*_1_, *∑*_2_, …, *∑*_*k*_) is the covariance vector, $Z={\left\{z_{n}\right\}}_{n=1}^{N}$ is the final integrated feature representation, *p*(*z*_*n*_) is the probability distribution function as a mixture of *K* Gaussian distributions. That is:

$$p\left(z_{n}\right)=\overset{K}{\underset{k=1}{\sum }}\pi_{k}p_{k}\left(z_{n}\right)=\overset{K}{\underset{k=1}{\sum }}\pi_{k}N\left(z_{n}|\mu_{k},\sum_{k}\right)$$ (12)

GMM used the EM algorithm to update the parameters *π*, *μ* and *∑*. According to the maximum probability density of the sample in different clusters, the most suitable subtype labels are obtained.

## 3 Experiments and analysis

### 3.1 Network structure and hyperparameter setting

The SADLN model has 19 layers, including 10 layers of the encoder, five layers of the decoder, and four layers of the discriminator. The specific network structure of SADLN is shown in Table 1. The model is built based on python 3.6.12, Keras 2.2.4, and TensorFlow 1.14.0 (the CPU version). The operating system is Windows 10. In terms of hardware, the CPU is Intel(R) Core (TM) i7-105 10U.

**TABLE 1:** The network structure of SADLN.

Architectures	SADLN	
Self-attention based encoder	3,105 + 3,217 + 383+3,139 (Input)	
	25 + 25+25 + 25 (concatenate)	
	100 (Batch normalization)	
	100 (Activation)	
	100 (Attention)	
	100 (Batch normalization)	
	100 (Fully-connected)	
	100 (Fully-connected, Mean)	100 (Fully-connected, VAR)
	100 (Output)	
Decoder	100 (Input)	
	100 (Fully-connected)	
	100 (Batch normalization)	
	100 (Activation)	
	3,105 + 3,217 + 383 + 3,139 (Output)	
Discriminator	100 (Input)	
	1 (Fully-connected)	
	1 (Sigmoid)	
	1 (Output)	

Optimizing hyperparameters are the key to training neural network models. Choosing appropriate hyperparameters can significantly improve the performance of the model. In this paper, the hyperparameters of the SADLN model mainly include the feature dimension of the independent sub network (d), the initial epoch, batch size, random seed, optimizer, activation function, learning rate and loss. Table 2 shows the hyperparameter settings of the SADLN model.

**TABLE 2:** Hyperparameter settings of SADLN model.

Hyperparameter	Setting
d	25
Epoch	600
Batch size	64
Random seed	2
Optimizer	Adam optimizer
Activation function	Gaussian error linear unit
Learning rate (lr)	1e-4, 2e-4, 3e-4, 4e-4, 5e-4, 1e-5, 2e-5, 3e-5, 4e-5, 5e-5
Loss *λ*_1_	1
Loss *λ*_2_	0.0001

### 3.2 Datasets and evaluation metrics

To evaluate the performance of our proposed method SADLN, we used ten TCGA cancers datasets provided by (Yang et al., 2021a) from https://github.com/haiyang1986/Subtype-GAN. The datasets include BRCA, LUAD, BLCA, PAAD, KIRC, STAD, UVM, GBM, SKCM, and UCEC. These ten datasets contain sufficient samples and have reasonable numbers of subtypes. There are four types of omics data for each cancer: copy number, DNA methylation, mRNA and miRNA. The datasets have been preprocessed and feature selection was performed. The preprocessing steps of four types data are as follows (Hoadley et al., 2018). The DNA methylation data were combined from two generations of Infinium arrays, HumanMethylation27 (HM27) and HumanMethylation450 (HM450). Firstly, the HM27 data against the HM450 data was normalized of 0–1 for *β*-values using a probe-by-probe proportional rescaling method. Then, 3,139 CpG sites were selected that were methylated at a *β*-value of ≥0.3 . For mRNA and miRNA data, firstly, the log transformation was performed separately, then poorly expressed genes were excluded based on median-normalized counts, and finally variance filtering was used to reduced features. Pre-processing led to 3,217 mRNA and 382 miRNA features. For copy number data, firstly, genomic regions along a chromosome defined by consecutive positions with a maximum Euclidean distance (based on copy number log-ratio segmented values) between any adjacent two probes smaller than 0.01 were formed; this resulted in a total of 3,105 copy number regions. Then each region was represented by its medoid signature, led to 3,105 copy number features. Finally, 3,105 copy number features, 3,217 mRNA features, 383 miRNA features and 3139 DNA methylation features were extracted from the original data source.

We used two evaluation metrics to evaluate the effect of cancer subtype recognition: survival analysis and clinical enrichment analysis. Survival analysis was obtained by the Cox log-rank test (Rainer and Muche and hosmer, 2001) to measure differential survival between subtypes. Smaller *p*-value indicates significant differences in survival profiles of different subtypes. In the clinical enrichment analysis, the differences in clinical indicators between subtypes were measured by the *p*-value obtained by Kruskal-Wallis test and Chi-square test for numerical and discrete clinical labels of cancer, respectively. Smaller *p*-value indicates significant differences between subtypes on this clinical label. Six clinical labels (Rappoport and Shamir, 2018) including age at diagnosis, gender, pathologic T, pathologic N, pathologic M, and pathologic stage were used for testing. The four latter parameters are discrete pathological parameters, measuring the size and extend of the primary tumor (T), the number of nearby lymph nodes that have cancer (N), whether the cancer has metastasized (M)and the total progression (pathologic stage). Cancer’s clinical parameters were not all available, such as GBM and UCEC only have two clinical parameters.

To avoid the influence of small cluster size on the accuracy of evaluation metrics, the permutation test (Rappoport and Shamir, 2018) was applied to calculate the *p*-value of Cox log-rank test in survival analysis and Chi-square test in clinical enrichment analysis. Permutation test obtains an empirical *p*-value using the test statistic by permuting the cluster labels between samples. To perform permutation tests, we randomly permuted the clustering assignments of the different samples. For the log-rank test, the number of permutations we performed for each clustering solution was first $\min\left(\left(\max\frac{10}{\text{original }p-value},1e4\right),1e6\right)$ and then another 1*e*5 permutations until the stopping condition was met. The stopping condition was having both the lower and upper ends of the 95% confidence interval for the *p*-value to be within 10% of its estimate, and such that the interval did not cross .05. For the clinical enrichment test, we continued on performing 1*e*3 permutations until the 95% confidence interval did not cross 0.05, up to a maximum of 1*e*5 iterations. This maximum number of iterations was only needed in case the *p*-value was extremely close to 0.05.

### 3.3 Ablation studies

To evaluate the contributions of key component of our model, we perform ablation studies in this section. There are three key modules in SADLN, self attention, decoder and discriminator. We separately removed these modules from SADLN, Table 3 gives the results of ablation studies in ten cancer datasets on TCGA.

**TABLE 3:** The −log10*p* values of ablation studies in ten cancer datasets on TCGA (bold indicates that this method performs best on the corresponding cancer dataset).

Cancer	SADLN	SADLN (NO SA)	SADLN (*λ*_2_ = 0)	SADLN (*λ*_1_ = 0)
BLCA	2.4	**2.5**	1.7	0.3
BRCA	**2.6**	2.3	0.4	0.4
GBM	**1.8**	1.7	0.4	0.2
KIRC	**6.6**	5.7	5.4	1.9
LUAD	**3.1**	2.4	0.3	0.04
PAAD	**3.2**	1.7	2.2	0.3
SKCM	3.0	**4.5**	0.9	0.8
STAD	1.4	1.3	**2.0**	0.3
UCEC	4.0	**5.4**	4.7	1.8
UVM	**4.5**	4.2	2.2	0.2

From Table 3, we can see that, compared with the model without the attention module, namely SADLN (NO SA), SADLN achieved better values on seven cancer datasets (BRCA, KIRC, GBM, LUAD, PAAD, STAD, and UVM). Compared with removing the discriminator module (*λ*_2_ = 0), SADLN obtained better value on eight cancer datasets (BRCA, GBM, KIRC, LUAD, PAAD, SKCM, UCEC, and UVM). The −log10*p* values of removing the decoder module (*λ*_1_ = 0) are lower than SADLN. These results indicates that three modules play an important role in addressing the issue of feature generation.

### 3.4 Comparison with other state-of-the-art algorithms

To verify the performance of SADLN, we compared it with ten state-of-the-art methods. Three deep learning based methods include AE, VAE and Subtype-GAN and seven non-deep learning based methods include K-means, LRAcluster, iCluster, Spectral, NEMO (Rappoport and Shamir, 2019), MCCA (Witten and Tibshirani, 2009) and SNF (Wang et al., 2014). These ten methods can represent different types of approaches for integrating multi-omics data. AE and VAE belong to early integration methods, both input and output are integrated multi-omics data. Subtype-GAN belong to middle integration method, the input and output are multi-omics features. For ten comparison algorithms, (Yang et al., 2021a) detailed the network structure, parameter selection and execution details its Supplementary Materials Note 1 and Note 2. In this study, we rigorously implement these algorithms following the guidelines of (Yang et al., 2021a).

To reduce the influence of different clustering numbers on the results of subtyping, following the work (Yang et al., 2021a), we set the cluster number of BRCA, LUAD, BLCA, PAAD, KIRC, STAD, UVM, GBM, SKCM and UCEC were 5, 3, 5, 2, 4, 3, 4, 4, 4, 4, respectively. These cluster numbers of different cancers have been proved to be clinically informed (Berger et al., 2018; The Cancer Genome Atlas Research Network, 2013; Robertson et al., 2017a; The Cancer Genome Atlas Research Network, 2014; Levine, 2013; Akbani et al., 2015; Li and Wang, 2021; Verhaak et al., 2010; Raphael et al., 2017; Robertson et al., 2017b). Table 4 gives the cluster number and subtypes of ten cancers. For example, in a previous study, GBM was classified into Classical, Mesenchymal, Neural, and Proneural subtypes based on mRNA expression data (Verhaak et al., 2010).

**TABLE 4:** The cluster number and subtypes of ten cancers.

Cancer	Cluster number	Subtypes
BRCA	5	LumA, LumB, Her2, Basal, Normal
LUAD	3	Terminal respiratory unit, Proximal inflammatory, Proximal proliferative
BLCA	5	Luminal-papillary, Luminal-infilitrated, Luminal, Basal/Squamous, Neuronal
PAAD	2	Basal-like/Squamous, Classical/Progenitor
KIRC	4	KIRC-M1, KIRC-M2, KIRC-M3, KIRC-M4
STAD	3	Immunity-Deprived (ImD), Stroma-Enriched (StE), Immunity-Enriched (ImE)
UVM	4	Disomy 3 (D3)-UVM-1, D3-UVM-2, Monosomy 3 (M3)-UVM-3, M3-UVM-4
GBM	4	Proneural, Neural, Classical, Mesenchymal
SKCM	4	Mutant BRAF, Mutant RAS, Mutant NF1, Triple-WT (wild-type)
UCEC	4	POLE (ultramutated), MSI (hypermutated), Copy-number high (serous-like), Copy-number low (endometrioid)

Table 5 gives the −log10*p* values of survival analysis for eleven methods of ten cancer datasets on TCGA. The clustering results of the other ten compared methods come from Yang’s literature (Yang et al., 2021a). Bold indicates that this method performs best on the corresponding cancer dataset.

**TABLE 5:** The −log10*p* values of survival analysis based on Cox log-rank model of ten cancers datasets on TCGA (bold indicates that this method performs best on the corresponding cancer dataset).

Cancer	SADLN	Subtype-GAN	AE	VAE	K-means	Spectral	LRA-cluster	SNF	NEMO	MCCA	iCluster
BLCA	2.4	**2.5**	0.1	0.1	0.6	1.8	0.1	1.2	2.3	1.1	1.0
BRCA	2.6	2.3	0.1	0.3	0.2	0.1	0.2	2.2	1.0	**2.7**	0.7
GBM	1.8	1.7	1.1	1.0	2.3	**2.6**	0.9	1.2	2.4	1.0	2.1
KIRC	6.6	5.7	2.6	6.0	4.2	4.6	**7.0**	4.4	4.3	7.0	3.9
LUAD	**3.1**	2.4	0.7	1.4	1.0	0.6	0.3	1.5	2.2	0.9	0.6
PAAD	**3.2**	1.7	0.1	2.5	2.3	2.0	2.2	2.1	2.0	2.1	1.0
SKCM	3.0	4.5	0.0	2.4	2.1	1.9	1.5	3.8	**4.7**	0.9	1.1
STAD	**1.4**	1.3	0.1	0.0	0.1	0.3	0.1	0.5	1.1	1.3	0.4
UCEC	4.0	5.4	0.4	5.4	5.7	0.8	4.2	5.0	**6.0**	5.0	1.3
UVM	**4.5**	4.2	2.7	2.1	1.6	1.9	2.3	2.5	2.3	2.4	1.1

As shown in Table 5, SADLN achieved the most significant results on PAAD, STAD, LUAD and UVM cancer datasets. Compared with Subtype-GAN, SADLN obtained better value on seven cancer datasets (BRCA, GBM, KIRC, LUAD, PAAD, STAD, and UVM). Compared with AE, SADLN obtained the best −log10p-value in ten cancer datasets. Compared with non-deep learning based methods, although same methods had best results in specific cancer datasets, the −log10*p*-value was highest on most cancer datasets.

Table 6 gives the clinical parameters enrichment analysis result of SADLN and other compared methods of ten cancer datasets.

**TABLE 6:** The clinical parameters enrichment analysis of SADLN and other methods of ten cancer datasets on TCGA (bold indicates that this method performs best on the corresponding cancer dataset).

Methods	BRCA	LUAD	BLCA	PAAD	KIRC	STAD	UVM	GBM	SKCM	UCEC
SADLN	5	3	5	2	**6**	**3**	0	**1**	1	**1**
Subtype-GAN	**6**	**5**	5	2	6	2	**2**	1	**4**	1
AE	0	1	0	1	5	1	0	1	0	0
VAE	5	2	**6**	1	6	2	1	0	1	1
K-means	5	1	3	0	6	2	0	1	1	1
Spectral	3	1	4	0	6	2	0	1	2	1
LRAcluster	5	1	3	1	6	1	0	0	0	1
SNF	5	3	6	2	4	1	0	0	4	1
NEMO	5	4	6	2	5	1	1	1	3	1
MCCA	5	4	3	**4**	3	2	1	1	0	1
iCluster	4	1	1	0	4	2	0	1	1	1

From Table 6, we can see that SADLN obtained the best results on four datasets (KIRC, GBM, STAD, UCEC). Therefore, we believe that SADLN is competitive with other methods in cancer subtype recognition.

**FIGURE 2:**
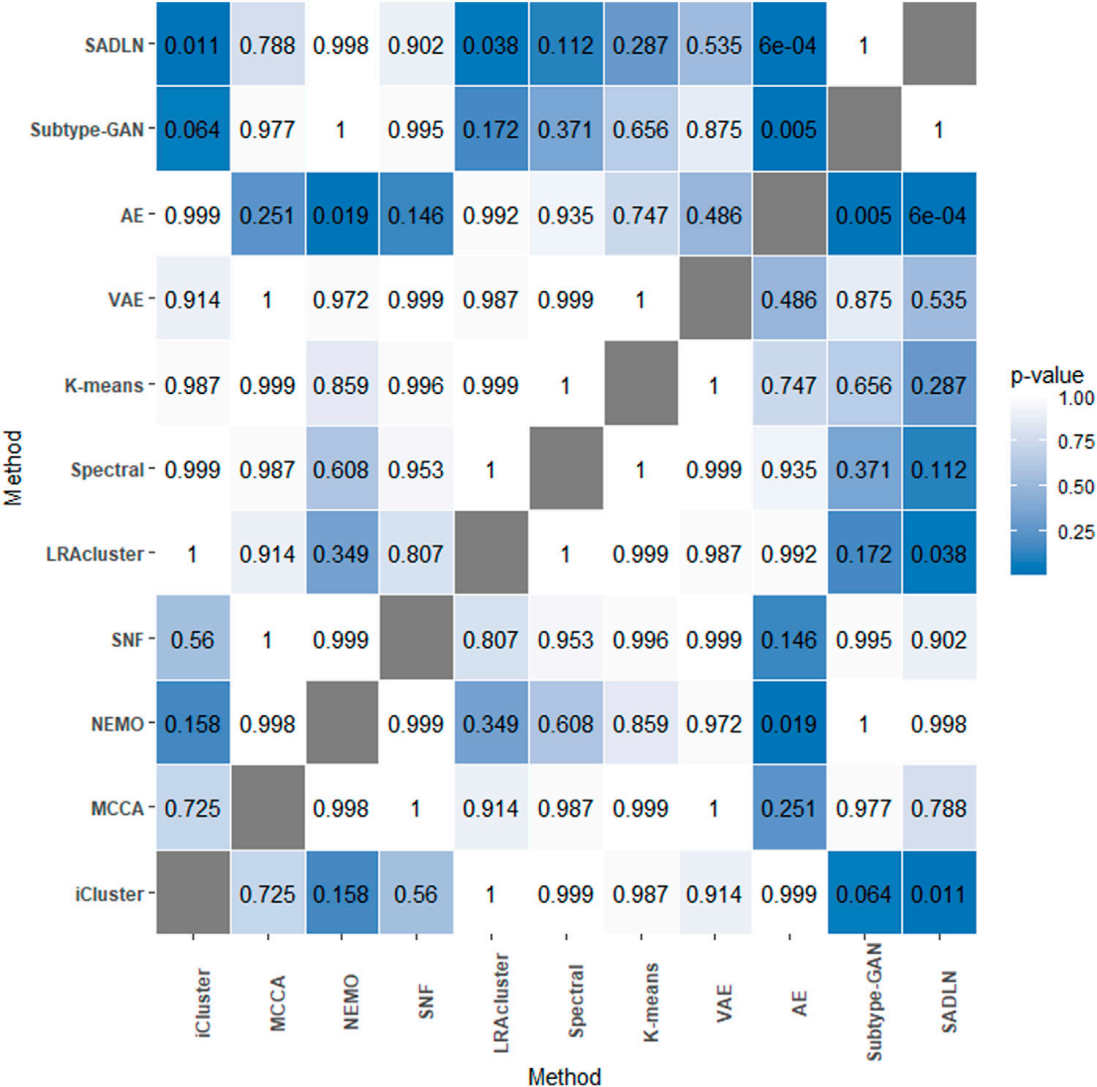
The *p*-values of the Friedman test on ten cancer datasets.

Friedman (1937) analysis was also used to evaluate the performance (Figure 2). From Figure 2, we can see that the performance of SADLN is better than the three methods iCluster, LRAcluster and AE (*p* < 0.05), but not better than other methods. We found that the performance of the methods is not exactly consistent under the two evaluation strategies.

### 3.5 Comparison of multiple omics data and single omics data

**FIGURE 3:**
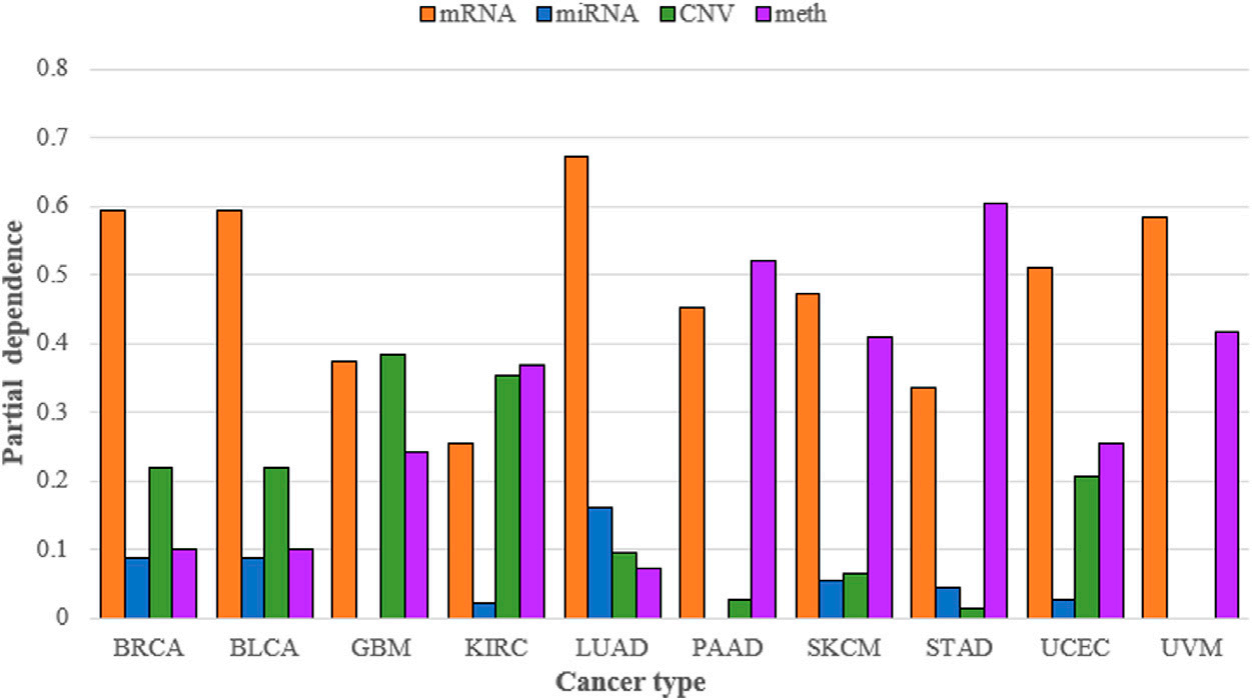
Contribution of mRNA, miRNA, CNV, and DNA methylation to the subtyping results of SADLN on ten cancer datasets.

SADLN integrated four types of omics data. To demonstrate the necessity of integrating multiple omics data for subtype recognition, we compared multiple omics data and single omics data of SADLN (denoted as SADLN-single) on subtyping results. We use the random forest (RF) method to analyze the contribution of different omics data on the subtyping results of SADLN. The input of RF is the four original omics features and the subtype labels of SADLN. The output of RF was the Gini importance scores of the features. We perform RF using scikit-learn (1.0.1) package of python, where the key parameter max_depth is set to six and the other parameters are set to the default values. We summed all the Gini importance scores belonging to each type of omics data and quantified the contribution of different omics data to the final subtyping results. The results are shown in Figure 3.

From Figure 3 we can see that the greatest contribution of BRCA, BLCA, LUAD, SKCM, UCEC, and UVM datasets was mRNA data, the greatest contribution of GBM was CNV data and the greatest contribution of KIRC, PAAD, and STAD was DNA methylation data. For different cancers, we choose the greatest contribution of omics data as the input of SADLN-single. The settings of parameters remain the same as SADLN. We also use the metric of *p*-value of survival analysis in Cox log-rank model to compare the performance of SADLN and SADLN-single (Table 7).

**TABLE 7:** The *p* values of survival analysis in Cox log-rank model of SADLN based multiple omics data and single omics data (bold indicates that this method performs better on the corresponding cancer dataset).

Cancer	SADLN	SADLN-single
BRCA	**2.40e-03**	4.23e-01
BLCA	**4.39e-03**	2.30e-02
LUAD	**7.69e-04**	3.00e-02
SKCM	**9.22e-04**	2.37e-01
STAD	**4.30e-02**	2.37e-01
UVM	**3.38e-05**	6.74e-01
GBM	**1.77e-02**	1.33e-01
KIRC	**2.77e-07**	1.74e-01
UCEC	**9.52e-05**	1.24e-01
PAAD	**6.39e-04**	1.40e-02

From Table 7, we can see that the *p*-values of SADLN are all smaller than the values of SADLN-single on ten cancer datasets. These results demonstrated that the integration of multiple omics data can help improve the performance of subtyping.

### 3.6 Survival analysis and visualization of clustering results

**FIGURE 4:**
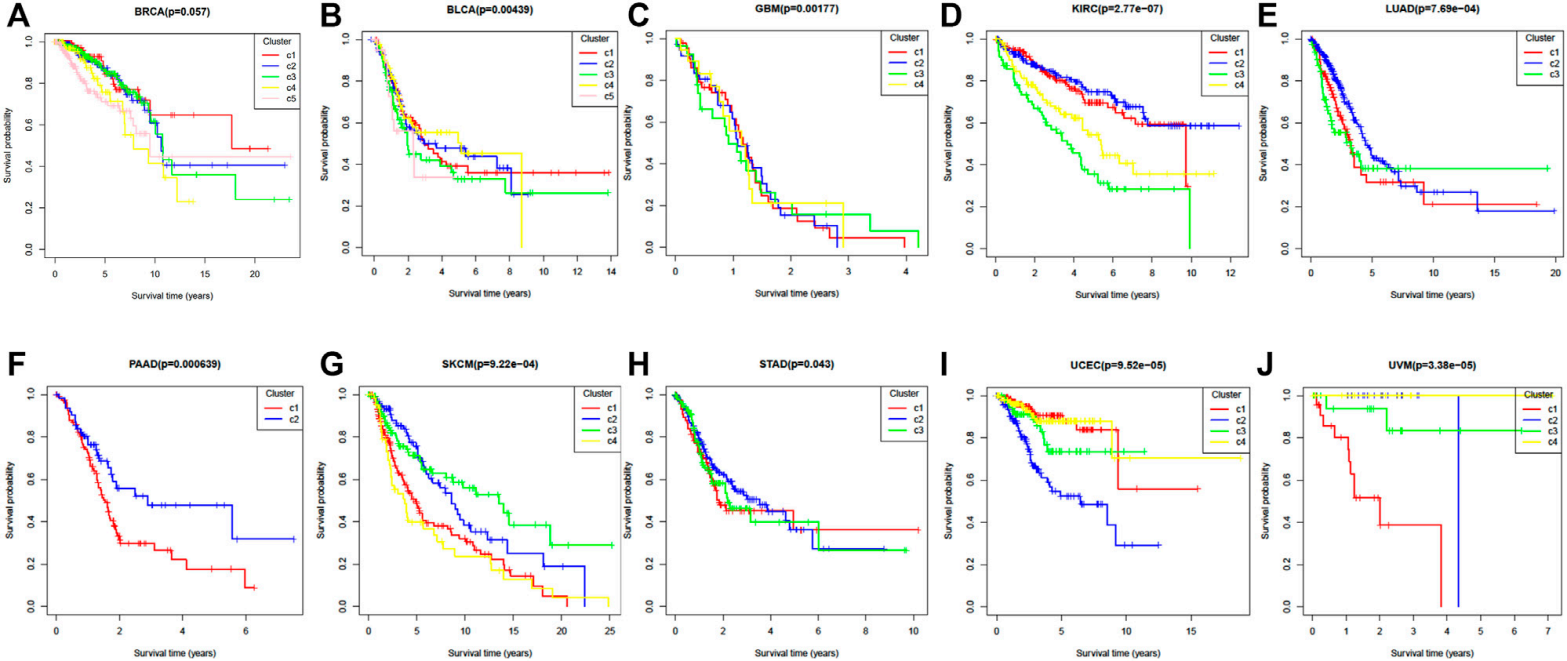
The Kaplan–Meier survival curves of ten cancer datasets. **(A)** BRCA, **(B)** BLCA, **(C)** GBM, **(D)** KIRC, **(E)** LUAD, **(F)** PAAD, **(G)** SKCM, **(H)** STAD, **(I)** UCEC, **(J)** UVM.

Survival curves can also be used to express the heterogeneity of different subtypes. Figures 4A–J shows the ten cancers’ Kaplan Meier survival alanalysis curves. From Figure 4, we can see that different clusters have significantly differences in survival curves (*p*-value < 0.05). Take BRCA cancer for example (Figure 4A), C1 has the longest average survival time, followed by C5, C2 and C3, C4 has a poor survival time.

**FIGURE 5:**
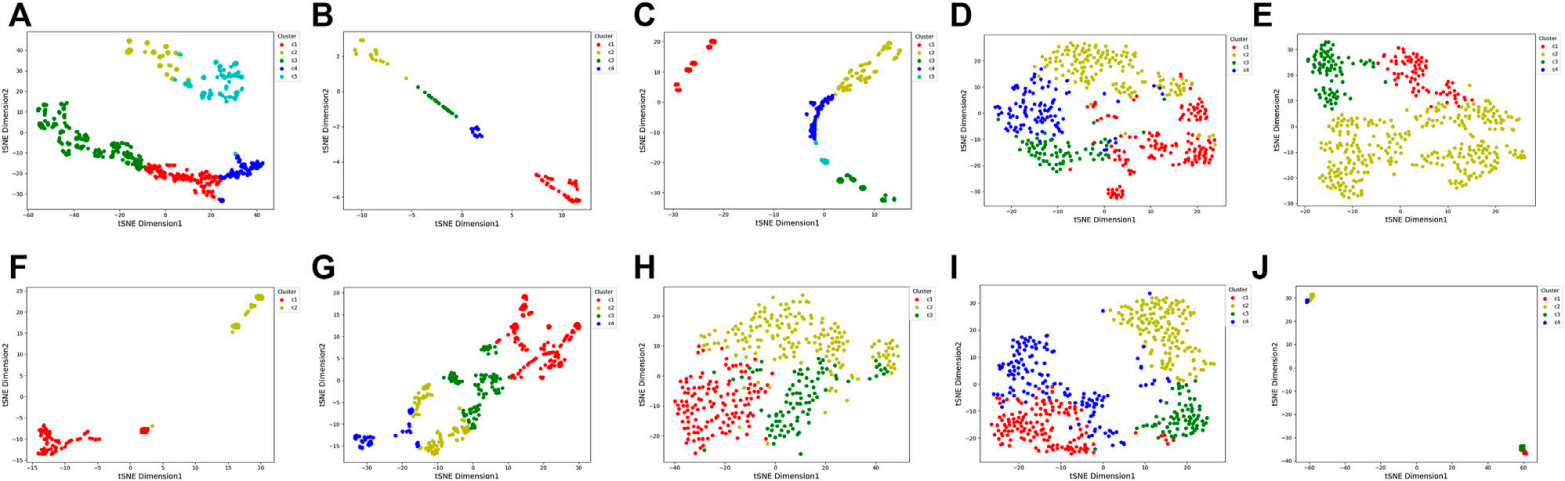
t-SNE visualization of the final integrated features by SADLN on ten cancer datasets. **(A)** BRCA, **(B)** BLCA, **(C)** GBM, **(D)** KIRC, **(E)** LUAD, **(F)** PAAD, **(G)** SKCM, **(H)** STAD, **(I)** UCEC, **(J)** UVM.

To visualize the clustering results, we used the t-SNE embedding method to display the final integrated feature representation of the SADLN (Figure 5). From Figure 5, we can see that samples of the same cluster are almost grouped together, and samples of different clusters are almost departed.

### 3.7 Case study

In this section, BRCA data is used to analyze the cancer subtypes obtained by the proposed method SADLN. Firstly, we analyzed the overlaps of the identified subtype clusters with the PAM50 cancer subtypes (Parker et al., 2009). There are five PAM50 cancer subtypes (Normal, LumA, LumB, Basal, and Her2), among 1,031 BRCA samples, only 803 samples have PAM50 subtypes including 128 Basal, 66 Her2, 405 LumA, 182 LumB, and 22 Normal. Table 8 shows the results of the overlap. From Table 8, we can see that, cluster C3 is enriched with LumA, of the 252 samples, 223 samples (88.49%) are LumA. Cluster C2 is enriched with LumA and LumB, of the 170 samples, 109 samples (64.12%) are LumA and 54 samples (31.76%) are LumB. Cluster C4 is enriched with LumB and LumA, of the 101 samples, 61 samples (60.40%) are LumB and 30 samples (29.70%) are LumA. Her2 and Basal samples are centrally distributed in clusters C1 and C5.

**TABLE 8:** The overlaps of the identified subtype clusters with PAM50 subtypes in BRCA cancer datasets.

Subtype ID	C1 (N = 134)	C2 (N = 170)	C3 (N = 252)	C4 (N = 101)	C5 (N = 146)
Basal (128)	60 (44.78%)	0 (0.00%)	0 (0.00%)	0 (0.00%)	68 (46.58%)
Her2(66)	26 (19.40%)	5 (2.94%)	2 (0.79%)	9 (8.91%)	24 (16.44%)
LumA (405)	20 (14.93%)	109 (64.12%)	223 (88.49%)	30 (29.70%)	23 (15.75%)
LumB (182)	20 (14.93%)	54 (31.76%)	17 (6.75%)	61 (60.40%)	30 (20.55%)
Normal (22)	8 (5.97%)	2 (1.18%)	10 (3.97%)	1 (0.99%)	1 (0.68%)

**FIGURE 6:**
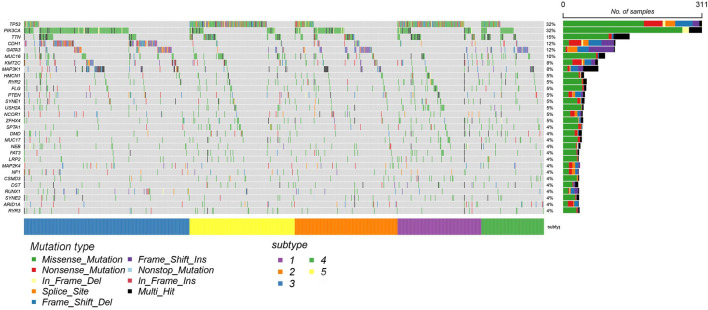
The mutation profiles of BRCA datasets with 20 significantly mutated genes using mRNA expression data.

In order to illustrate the difference between the identified subtype clusters of SADLN, we also analyzed the mutation profiles of BRCA using mutation data (the mutation data can be found at https://portal.gdc.cancer.gov). Among 1,031 samples in BRCA datasets, 820 samples have the mutation data. Figure 6 gives the 20 significantly mutated genes of the identified subtype clusters. From Figure 6, we can see that, clusters C2 and C3 have a significant difference in the frequency of PIK3CA and CDH1 genes, although clusters C2 and C3 are all dominated by LumA subtype. The C1 and C5 clusters have a high frequency of TP53 gene mutations, this also explains why clusters C1 and C5 are dominated by Basal and Her2 subtypes.

**FIGURE 7:**
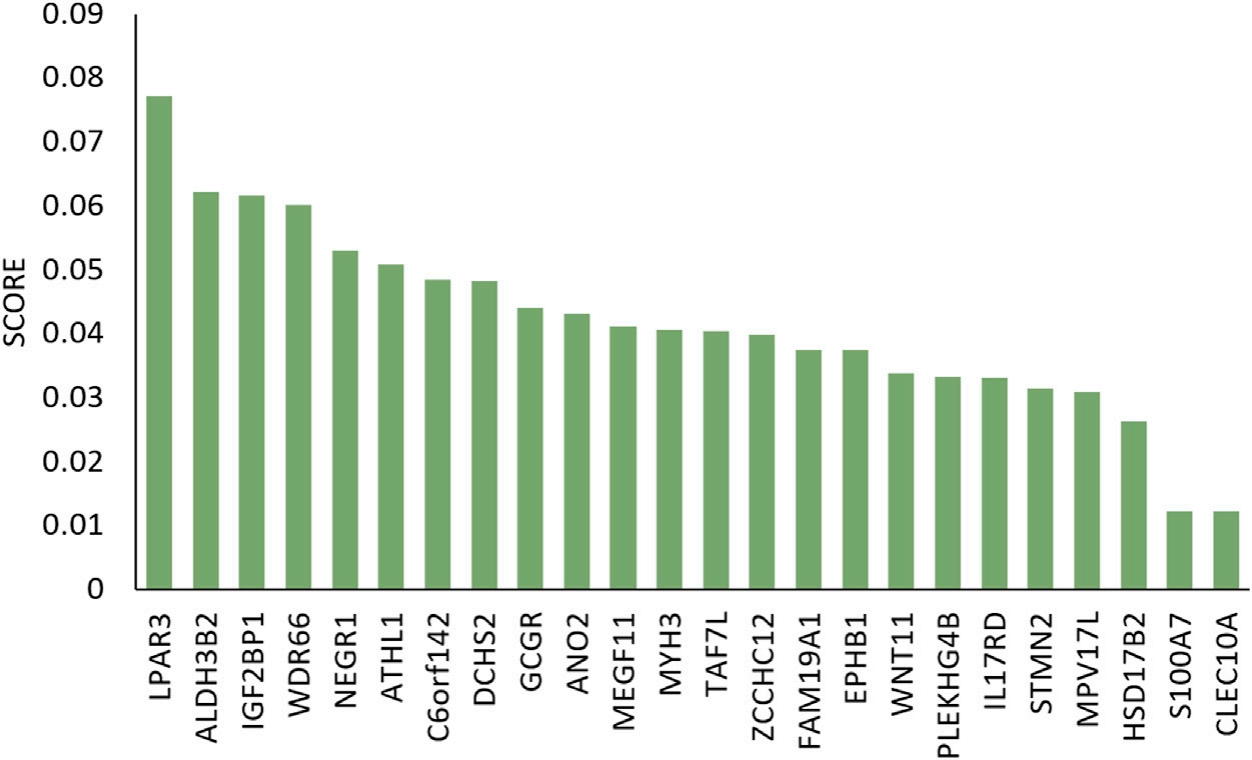
Gini importance scores of differential genes in C1 and C5 clusters.

To illustrate the difference between clusters C1 and C5, we used RF method to analyzed the differential genes using mRNA expression data. Figure 7 gives the result.

Among these differential expression data, study has shown that the expression of ALDH3B2 was higher in SK-BR-3 cells compared with in other subtypes of breast cell lines, as determined by reverse transcription-polymerase chain reaction and western blot analysis. In addition, the expression levels of ALDH3B2 were higher in Her2 positive breast cancer compared with in other subtypes of breast cancer, as determined by immunohistochemistry, which may be used as a prognostic indicator for breast cancer (Feng et al., 2019). The expression level of CLEC10A to be positively associated with the level of different tumor-infiltrating immune cells in BRCA including CD8 T cells, B cells, macrophages, and NK cells. These results suggest that the relationship between lower CLEC10A expression level and poor prognosis in BRCA may be due to the role of CLEC10A in the tumor immune microenvironment (Tang et al., 2022).

### 3.8 Identify the key biomarkers in each cancer

To identify the key biomarkers that determine the subtyping results in each cancer, we ranked the importance of mRNA features of each cancer dataset using the clustering labels of SADLN and RF method to achieve the five most essential biomarkers. For each cancer, Table 9 gives the five biomarkers most relevant to ten cancers.

**TABLE 9:** The five biomarkers most relevant to ten cancers.

Cancers	Biomarkers
BRCA	AGR3, GDF10, EEF1A2, ATP6V0A4, GIPC2
BLCA	GFPT2, SNX31, RASSF9, MUC4, CACNG3
GBM	SHROOM3, PLEKHG4B, CNTNAP4, KCP, PEG10
KIRC	ITPKA, PTPN3, SEMA3B, LRRC55, DNASE1L3
LUAD	HPGDS, UGT1A4, C1orf116, STAT4, ZG16
PAAD	RIMSI, HAL, PAX8, THEM5, EDN2
SKCM	PGLYRP3, TFAP2A, IGSF3, COL17A1, OGDHL
STAD	MEOX2, LIMS2, BEND7, TPSG1, APLN
UCEC	SPDEF, SORBS2, C1orf192, CD163L1, BCL2L14
UVM	PYGM, SCNN1A, SERPINA3, SLC47A22, PRPH

For BRCA as an example, the five key biomarkers are AGR3, GDF10, EEF1A2, ATP6V0A4, and GIPC2. By literature review, we found that the AGR3 gene (de Moraes et al., 2022) affects the prognosis of luminal breast cancer patients. EEF1A2 gene (Hassan et al., 2020) and the GDF10 (Zhou et al., 2019) gene have influenced the prognosis of triple-negative breast cancer patients. The study has shown that the expression of the ATP6V0A4 gene (Savci-Heijink et al., 2019) is a signature of visceral organ metastasis in breast cancer. Although the GIPC2 gene (Dong et al., 2021) has not been found in BRCA but has been shown that it acts on the pathogenesis and development of a pheochromocytoma. All these literature reviews demonstrated the results of SADLN on the BRCA dataset are reliable.

## 4 Discussion

Recently, integrating multi-omics data for cancer subtyping is an important task in bioinformatics. In this paper, we proposed SADLN, a novel deep learning based integrated method for cancer subtyping. The method firstly introduced self-attention into the encoder-decoder based network architecture. It attempted to describe complex and diverse multi-omics data accurately and adaptively build the samples’ relationship when learning a shared low-dimensional representation during molecular subtyping. Compared with three deep learning and seven non-deep learning based integration algorithms, SADLN has two characteristics: 1) Unlike the early integration methods such as AE and VAE, SADLN characterizes multi-omics data respectively which enables the model to effectively describe different omics data with distinct distributions, meanwhile, the output integrating representation fits the prior distribution. 2) The self-attention module in SADLN taking full use of the sample’s multi-omics information, can automatically learn the weight matrix between samples and make the results of feature integration more convincing.

We demonstrated the power of SADLN using ten datasets of TCGA. The experiments of survival analysis and Friedman analysis show that SADLN has a good clustering consequence. Meanwhile, the experiments of SADLN and SADLN-single show that integrating multiple omics data is a necessity and useful. The BRCA results indicated that SADLN can efficiently distinguish cancer subtypes.

SADLN found 50 biomarkers for all cancers. Some biomarkers have been verified in previous studies. In clinical research, researchers can conduct more subtype analysis studies on related cancers based on the biomarkers obtained by SADLN. For example, SADLN believes that MEOX2 is an important biomarker of STAD. The study (Wang et al., 2021) has shown that MEOX2 is a novel biomarker associated with macrophage infiltration in digestive system cancer.

Although SADLN has enhanced the performance of cancer subtyping recognition, it also has limitations. Firstly, it is unsuited to integrate binary data. Secondly, it could not find the genes modules that affect each subtype. Thirdly, the relationship between omics data was not considered. For the next research, we will continue our efforts to develop an attention based method to simultaneously learn the relationship between multi-omic and samples to explore cancer heterogeneity.

## 5 Conclusion

In this paper, we proposed Self-Attention Based Deep Learning Network (SADLN) for integrating multi-omics data for cancer subtype recognition. The novel method is based on recent advances in deep learning and self-attention. It can jointly learn different multi-omic data representations and relations between samples. In comparison to the state-of-the-art methods, experiments on ten datasets of TCGA have demonstrated the effectiveness of SADLN.

## Data Availability

The original contributions presented in the study are included in the article/supplementary material, further inquiries can be directed to the corresponding author.
